# Surface diagnosticity predicts the high-level representation of regular and irregular object shape in human vision

**DOI:** 10.3758/s13414-019-01698-4

**Published:** 2019-03-12

**Authors:** Irene Reppa, E. Charles Leek

**Affiliations:** 1 Department of Psychology, Swansea University, Swansea, SA2 8PP UK; 2 School of Psychology, Institute of Life and Human Sciences, University of Liverpool, Liverpool, UK

**Keywords:** Visual perception, Object recognition, Shape regularity, Surface diagnosticity, Priming, Whole-part matching

## Abstract

The human visual system has an extraordinary capacity to compute three-dimensional (3D) shape structure for both geometrically regular and irregular objects. The goal of this study was to shed new light on the underlying representational structures that support this ability. Observers (*N* = 85) completed two complementary perceptual tasks. Experiment 1 involved whole–part matching of image parts to whole geometrically regular and irregular novel object shapes. Image parts comprised either regions of edge contour, volumetric parts, or surfaces. Performance was better for irregular than for regular objects and interacted with part type: volumes yielded better matching performance than surfaces for regular but not for irregular objects. The basis for this effect was further explored in Experiment 2, which used implicit part–whole repetition priming. Here, we orthogonally manipulated shape regularity and a new factor of surface diagnosticity (how predictive a single surface is of object identity). The results showed that surface diagnosticity, not object shape regularity, determined the differential processing of volumes and surfaces. Regardless of shape regularity, objects with low surface diagnosticity were better primed by volumes than by surfaces. In contrast, objects with high surface diagnosticity showed the opposite pattern. These findings are the first to show that surface diagnosticity plays a fundamental role in object recognition. We propose that surface-based shape primitives—rather than volumetric parts—underlie the derivation of 3D object shape in human vision.

The human vision system is remarkable for its ability to process sensory information about the shapes of three-dimensional (3D) objects. The perception of shape underpins our ability to recognise objects, to understand scene content, and to interact with the environment. It is now widely accepted that shape perception starts with the detection of edges from changes in luminance intensity in V1 (e.g., Hubel & Wiesel, 1962), and the derivation of increasingly abstract position and scale-invariant shape features in higher visual areas. This general processing architecture is reflected most directly in recent hierarchical models of image classification based on biologically inspired deep networks (e.g., Kheradpisheh, Ghodrati, Ganjtabesh, & Masquelier, 2016; Krizhevsky, Sutskever, & Hinton, 2012; LeCun, Bengio, & Hinton, 2015; Riesenhuber & Poggio, 1999; Serre, Oliva, & Poggio, 2007; Serre, Wolf, Bileschi, Riesenhuber, & Poggio, 2007). However, despite these advances, our understanding of the organisation and structure of higher-order shape representations remains relatively poor. Pizlo and colleagues (e.g., Pizlo, Sawada, Li, Kropatsch, & Steinman, 2010; Sawada, Li, & Pizlo, 2011) have recently shown that veridical representations of 3D shape can be recovered from a single two-dimensional (2D) view of an edge-based input image when the derivation follows a priori simplicity constraints based on symmetry and volume. At the same time, other evidence suggests that the representation of 3D shape also involves the derivation of other kinds of (higher-order) geometric properties of shape (e.g., Barr, 1981; Bergevin & Levine, 1993; Biederman, 1987; Biederman & Cooper, 1991; Guzman, 1968; Krivic & Solina, 2004; Marr & Nishihara, 1978; Pentland, 1986; Ullman, Vidal-Naquet, & Sali, 2002; Zerroug & Nevatia, 1999). These primitives include 2D geons (e.g., Biederman, 1987), surfaces (e.g., Faugeras, 1984; Fisher, 1989; Leek, Reppa, & Arguin, 2005; Leek, Reppa, Rodriguez, & Arguin, 2009; Leek, Roberts, Dundon, & Pegna, 2018; Leek, Roberts, Oliver, Cristino, & Pegna, 2016; Marr & Nishihara, 1978; Reppa, Greville, & Leek, 2015), and volumetric primitives, such as 3D geons (e.g., Biederman, 1987), generalized cylinders (e.g., Brooks, 1981; Marr & Nishihara, 1978), and superquadrics (e.g., Barr, 1981; Pentland, 1986).

Surfaces, as a higher-order primitive, have been shown to play a key role in visual perception (e.g., Cate & Behrmann, 2010; Norman & Todd, 1996; Norman, Todd, Norman, Clayton, & McBride, 2006; Norman, Todd, & Phillips, 1995). They can influence facilitatory and inhibitory components of attention (e.g., Leek, Reppa, & Tipper, 2003; Nakayama, He, & Shimojo, 1995; Nakayama & Shimojo, 1992; Reppa & Leek, 2003, 2006; Reppa, Schmidt, & Leek, 2012). For example, Leek et al. (2005) have shown that response latencies to match stimuli comprising subsets of whole object contours decreased for stimuli corresponding to spatially adjacent object surfaces compared with perceptually closed, but not surface-grouped, contours. More recently, using event-related potentials (ERPs), Leek et al. (2018; Leek et al., 2016) have found evidence of differential early perceptual sensitivity to higher-order surface and volumetric part structure within the first 200 ms of shape perception. These findings suggest that the perception of 3D object shape can involve the derivation of higher-order surface structure.

One issue that has received little attention in previous work is how geometric regularity may influence the kinds of representations that are computed during shape perception (e.g., Kayaert, Biederman, & Vogels, 2004; Kimia, 2003; Pizlo et al., 2010). Geometric regularity can be defined by the presence of mirror and/or translational symmetry, and concomitant shape redundancy (e.g., Pizlo et al., 2010). Regular 3D objects can be characterized in terms of predictability of the nonvisible surfaces (i.e., perceptual completion of the rear of an object can be implied by the completion of the front of the object; e.g., van Lier & Wagemans, 1999). Most prior studies, even those using novel object sets, have been based on geometrically regular shape (e.g., Biederman, 1987; Biederman, Kayaert, & Vogels, 2004; Leek et al., 2005; Leek et al., 2009; Leek et al., 2018; Leek et al., 2016; Reppa et al., 2015). Relatively little is known about the representation of geometrically irregular objects, despite the fact that the visual system has the flexibility and capacity to represent irregular object geometry, such as that found in many generally naturally occurring, and frequently encountered, forms (e.g., rocks).

Understanding how human vision processes irregular object shape provides an opportunity to gain new insights into the representational structure or structures underlying its adaptive flexibility. Furthermore, it remains unclear how well empirical findings from previous work using geometrically regular objects generalise to irregular forms. In several theoretical models, symmetry is attributed a fundamental role in the recovery of 3D shape volume—for example, as an a priori simplicity constraint (e.g., Pizlo et al., 2010; Sawada et al., 2011), or as a key factor in the perceptual grouping of low-level image features (e.g., Machilsen, Pauwels, & Wagemans, 2009; Wagemans, 1995), and in the decomposition of 3D shape into constituent higher-order shape properties including surfaces and volumetric parts within the context of structural description models of shape representation (e.g., Biederman, 1987; Hoffman & Richards, 1984; Marr & Nishihara, 1978). Thus, it may be expected that the presence or absence of symmetry in geometrically regular or irregular 3D objects can influence the kinds of higher-order shape information that are computed during shape perception. For example, the absence of symmetry or skewed symmetry in the 2D (retinal) projection of a geometrically irregular 3D object may reduce, or render difficult, the recovery of volumetric structure, and increase reliance on surface shape. On this basis, one might predict an interaction between geometric regularity and the underlying representation of intermediate shape structure.

## Experiment 1

**Fig. 1:**
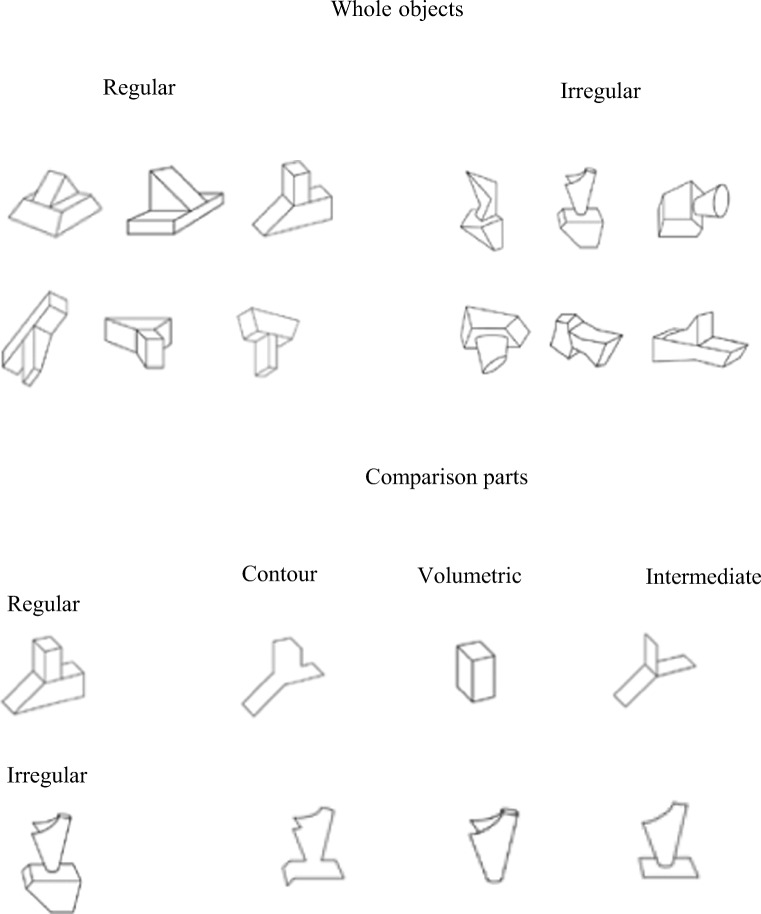
Illustration of the 12 objects (six regular and six irregular) used in Experiment 1, and examples of the comparison parts for object type (see Experiment 1, Method, for details)

Experiment 1 examined whether geometric regularity modulates sensitivity to volumetric and/or surface shape structure in a modified variant of the whole–part matching paradigm used by Leek et al. (2005). As in Leek et al. (2005, Experiment 3), matching performance was compared between three types of comparison part. *Closed-contour* parts consisted of segments of object-internal and bounding contour. *Volumetric parts* consisted of one of two constituent volumetric components, while *intermediate parts* consisted of adjacent surfaces that did not make up a complete volume (see Fig. 1). In addition, shape regularity was manipulated. The whole object stimulus set comprised both geometrically regular and irregular novel shapes. Regularity was defined by the presence or absence of mirror and/or translational symmetry (e.g., Li, Sawada, Shi, Steinman, & Pizlo, 2013). On the hypothesis that the presence of symmetry is fundamental for the recovery of volumetric structure, an advantage was predicted for the whole–part matching of volumetric parts for geometrically regular objects, but not for irregular objects—that is, an interaction between shape regularity and part type was expected.

### Method

#### Participants

Forty-five adult students and research assistants in the School of Psychology, Bangor University (*M*_age_ = 28 years, *SD* = 8.0) participated for £3.00 or for course credits. An a priori power analysis indicated that approximately 42 participants would be needed to detect a medium-sized effect when employing the traditional .05 criterion of statistical significance. All participants reported normal or corrected-to-normal vision.

#### Apparatus and stimuli

The experiment was controlled by PsychLab (Gum, 1995) run on an Apple Macintosh G4 computer, and stimuli were displayed on a 17-inch RGB monitor at a viewing distance of 57 cm.

Twelve opaque black-and-white line drawings of novel three-dimensional objects were used. Each fitted within a 6 × 6 cm frame (not visible during the experiment) subtending 6.2^o^ (see Fig. 1). The stimuli were created by hand using Adobe Photoshop. Every effort was made to avoid creating objects that might look like familiar objects, and this was confirmed among the authors.

Each object consisted of one larger and one smaller volumetric component.

Six objects were composed of two geometrically regular components, and six were composed of two geometrically irregular components (see the Introduction for definitions of regular and irregular components). In regular objects (see Fig. 1, top left), both components had bilateral symmetry and predictable shape of the self-occluded part of the object—that is, the shape of the part of the object that was not visible could be reliably predicted on the basis of the visible shape of the objects. In contrast, in irregular objects (see Fig. 1, top right), neither component had mirror symmetry, and the self-occluded parts of the object could not be reliably predicted on the basis of the visible object shape.

For each object, three types of comparison stimuli were created, shown in Fig. 1 (bottom panel): closed contour, intermediate, and volumetric. The volumetric parts were either of the two components of the object. The closed contour component parts were made by deleting regions of object contour with the constraint that the resulting image was a closed form that did not correspond to any complete object surface. The specific contours chosen were decided based on whether the resulting edge contour matched in terms of length the edge contour of the volumetric parts as closely as possible. Finally, the intermediate parts consisted of the same number of surfaces (closed regions that correspond to object surfaces) as the volumetric parts but the surfaces did not form a volume. Apart from this constraint the surfaces chosen to be part of the intermediate primes depended on how closely the resulting edge contour of the intermediate part for a specific object matched that of the volumetric parts of the same object. In our previous work, we have referred to the intermediate parts as *surface* parts (e.g., Leek et al., 2005; Leek et al., 2009). However, here we opted to use the term *intermediate* in order to avoid confusion with another experimental condition, which was relevant in Experiment 2.

To prevent contour overlap between the whole object and the comparison parts, the whole-object stimuli were enlarged by 150% of their original size.

#### Summary of low-level features

Table 1 shows low-level image properties for each comparison part: mean percentage of edge contour, bounding contour, and number of vertices (L and Y).

**Table 1:** Description of low-level properties of the contour, volumetric, and surface parts used in Experiment 1 for the six regular and six irregular objects

	Part type	Edge contour (%)	Bounding contour (%)	*N* vertices				Surface diagnosticity value
				L	Y	T	% vertex change	
		Mean (*SD*)	Mean (*SD*)	Mean (*SD*)	Mean (*SD*)	Mean (*SD*)	Mean (*SD*)	Mean (*SD*)
Regularity								
Regular	Contour	42.47 (5.09)	50.56 (11.60)	7.66 (2.25)	N/A	N/A	71.13 (20.08)	N/A
	Volumetric	42.72 (3.38)	33.98 (4.80)	3.00 (.60)	3.33 (1.03)	2.33 (1.21)	20.24 (17.15)	.84 (.02)
	Intermediate	47.38 (7.70)	41.48 (10.49)	4.67 (1.21)	2.83 (0.82)	2.50 (.84)	53.66 (13.91)	.83 (.02)
Irregular	Contour	49.13 (4.01)	61.33 (11.71)	10.00 (2.09)	N/A	N/A	72.73 (11.86)	N/A
	Volumetric	41.32 (9.26)	39.10 (11.30)	4.00 (1.67)	2.33 (.82)	2.50 (.88)	3.70 (9.07)	.95 (.04)
	Intermediate	52.04 (3.19)	37.27 (9.08)	7.67 (1.51)	1.00 (1.26)	3.17 (.75)	42.00 (14.89)	.94 (.04)

In the N/A cells there are no corresponding values

##### Edge contour

Regular and irregular comparison parts did not differ in the percentage of total edge contour they contained, *t*(10) = 1.25, *p* >.05. For regular objects, there were no significant differences in percentage of edge contour between contour, volumetric, and intermediate parts, *t*(5) < 1and *p* > .05, in all cases. For irregular objects, contour parts did not differ from intermediate parts, *t*(5) = 2.16, *p* > .05. However, volumetric parts had less edge contour than both contour and intermediate parts, *t*(5) = 2.49, *p* = .03 and *t*(5) = 4.16, *p* < .001, respectively.

##### Bounding contour

Bounding contour can give information about the global shape of the stimulus (e.g., Hayward, 1998; Lloyd-Jones & Luckhurst, 2002). We calculated how much contour in each comparison part came from the perimeter of each whole object and expressed it as a percentage of the entire edge contour of each part type. This further allowed us to control for the fact that contour parts, as well as intermediate and volumetric parts contain edges with different contour semantics—that is, some edges came from the object’s bounding contour while others were surface discontinuities (see Mooney, 1957; Rubin, 2001, for discussion). There was no overall difference in bounding contour between regular and irregular parts, *t*(10) < 1, *p* > .05*.* For regular objects, contour parts contained more bounding contour than did volumetric parts, *t*(5) = 4.14, *p* < .01, but there was no difference between volumetric and intermediate parts, *t*(5) = 1.96, *p* > .05, and *t*(5) = 2.04, *p* > .05, respectively. For irregular objects, contour parts also contained more bounding contour than volumetric, *t*(5) = 6.12, *p* < .01, and intermediate parts, *t*(5) = 3.41, *p* < .05. There was no difference in bounding contour between irregular intermediate and volumetric parts, *t*(5) < 1, *p* > .05.

##### Vertices

Previous literature has shown that intersections, such as L vertices; Y, or fork, vertices; and T, or arrow, vertices can be particularly informative about the object shape and the spatial configuration of its parts, and self-occlusion (e.g., Biederman, 1987; Lowe, 2003). Irregular parts overall contained more L and Y vertices than regular parts, *t*(10) = 3.30, *p* < .01, and *t*(10) = 2.49, *p* < .05, respectively, but no difference in the number of T vertices, *t*(10) < 1, *p* > .05. For regular objects, contour parts contained more L vertices than did volumetric and intermediate parts, *t*(5) = 5.08, *p* = .01, and *t*(5) = 2.90, *p* = 03, respectively. Intermediate parts contained significantly more L vertices than volumetric parts, *t*(5) = 3.37, *p* = .04, but did not differ in terms of number of Y vertices, *t*(5) = 1.46, *p* > .05 or T vertices, *t*(5) < 1, *p* > .05. Regular volumetric parts had a lower proportion of vertex change than both contour parts, *t*(5) = 4.86, *p* = .01, and intermediate parts, *t*(5) = 3.07, *p* = .03.

For irregular objects, volumetric parts contained significantly fewer L vertices than did both contour and intermediate parts, *t*(5) = 8.78, *p* < .001, and *t*(5) = 3.99, *p* = .01, respectively, whilst there was no difference between contour and intermediate parts, *t*(5) = 2.54, *p* > .05. Irregular volumetric parts contained more Y vertices than did irregular intermediate parts, *t*(5) *=* 3.50, *p* = .03, but fewer T vertices than did intermediate parts, *t*(5) = 3.16, *p* = .05. There were no Y or T vertices in the contour parts. A greater proportion of vertices changed from one type to another (e.g., from Y or T to L) for contour parts compared with volumetric parts, *t*(5) = 12.67, *p* < .001, and compared with intermediate parts, *t*(5) = 4.02, *p* = .01. There was a greater vertex change for surface compared with volumetric parts, *t*(5) = 8.21, *p* < .001.

#### Design

The experiment was based on a 2 (matching: match vs. mismatch) × 2 (regularity: regular vs. irregular) × 3 (part type: contour, volumetric, intermediate parts) within-participants design, yielding 12 experimental conditions. There were 288 trials plus 12 practice trials. The trials were split into four equal blocks of 72 trials, within which all trials were randomized. The dependent measures were response times and accuracy.

#### Procedure

**Fig. 2:**
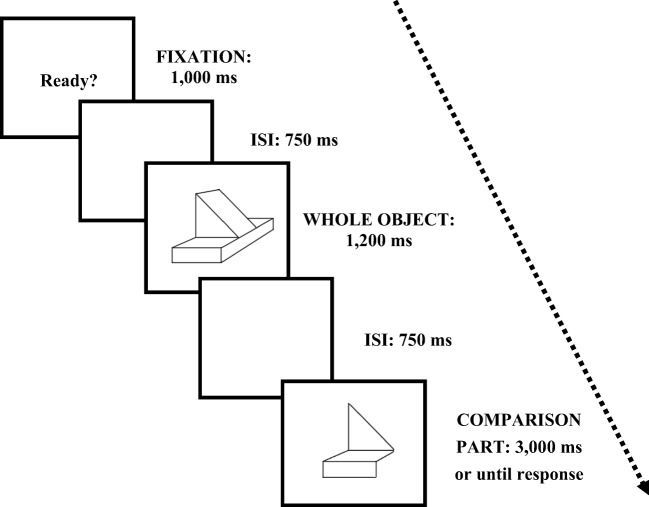
Example of a match trial for a regular object and its intermediate comparison part, in Experiment 1

The sequence of events in a trial is depicted in Fig. 2. Each trial started with a 1^o ×^ 1^o^ fixation cross at screen centre for 1,000 ms. After a blank 750 ms interstimulus interval, one of the whole objects appeared at screen centre for 1,200 ms. Finally, following a blank interval of 750 ms, the comparison stimulus appeared for 5,000 ms or until response. Participants had to decide as fast and accurately as possible whether the comparison part came from the whole object or not. Incorrect responses or time-outs were signalled with a ‘beep’ and an ‘Incorrect’ message on the screen. Responses were made through the keys *D* and *K* for yes and no responses, respectively, for half of the participants, and the assignment was reversed for the other half.

### Results

Incorrect responses (*M* = 19.45%, *SD* = 7.5%) were removed from the data and analysed separately. Correct response times (RT) were trimmed to ± 2 standard deviations from the mean per condition, which led to the removal of 3.9% from the total number of trials.

#### Response times (RT)

**Fig. 3:**
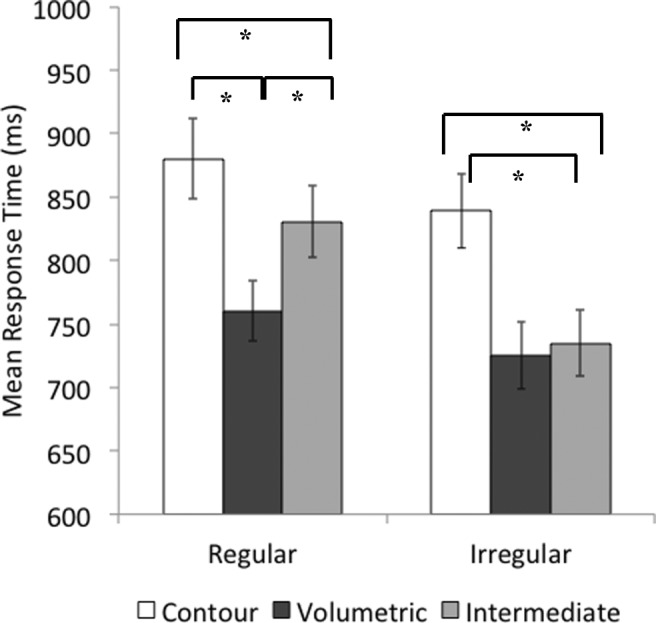
Mean response times (milliseconds) per condition for regular and irregular objects in match trials in Experiment 1. Error bars show standard error of the mean

Analysis of RT was carried out on correct responses of the match trials. Cell means are shown in Fig. 3.

A 2 (regularity: regular, irregular) × 3 (part type: contour, volumetric, intermediate) repeated-measures ANOVA showed significant main effects of regularity, *F*(1, 44) = 25.88, *p* < .001, η_p_^2^ =.37, with regular RT slower than irregular RT, and part type, *F*(2, 88) = 31.08, *p*< .001, η_p_^2^ =.41, with contour parts leading to slower matching times compared with volumetric and intermediate parts, with no difference between that latter two conditions. The Regularity × Part Type interaction was significant, *F*(2, 88) = 4.27, *p* < .05, η_p_^2^ =.09.

Planned contrasts to examine the interaction revealed that for regular objects, matching was slower for contour parts compared with both volumetric and intermediate parts, *t*(44) = 4.77, *p* < .001, and *t*(44) = 2.26, *p* < .05, respectively. Regular volumetric parts were matched faster than were intermediate parts, *t*(44) = 2.97, *p* = .005. The pattern for irregular objects was different. As with regular objects, matching for contour parts was slower than both volumetric and intermediate parts, *t*(44) = 6.84, *p* < .001, and *t*(44) = 5.55, *p* < .001, respectively. However, the speed of matching irregular volumetric and intermediate parts did not differ, *t*(44) = 0.36, *p* > .05.

#### Analyses of significant image properties on RTs

For each of the dimensions in which image properties differed significantly, tests were performed to determine the strength of the relationship between the image dimension and observed RT. Irregular volumetric and intermediate parts differed in the mean percentage of bounding contour, and correlations showed that the mean amount of edge contour did not correlate significantly with RTs for regular (*r*^2^ = .03, *p* > .05), or for irregular objects (*r*^2^ = .02, *p* > .05). Volumetric parts contained significantly fewer L vertices than intermediate parts for both regular and irregular objects, and significantly more Y vertices and significantly fewer T vertices than intermediate parts for irregular objects only. The correlation between L, Y, and T vertices and RTs was not significant for either regular (*r*^2^ < .1, *p* > .05, for both types of vertex) or for irregular objects (*r*^2^ < .1, *p* > .05, for all three types of vertex). Finally, for irregular objects only there was a larger proportion of vertex change for intermediate than volumetric parts, and there was no significant correlation between proportion of vertex change and mean RT (*r*^2^ < .1, *p* > .05). These results suggest the pattern of differences in RT cannot be accounted for by low-level image differences.

#### Error rates

**Fig. 4:**
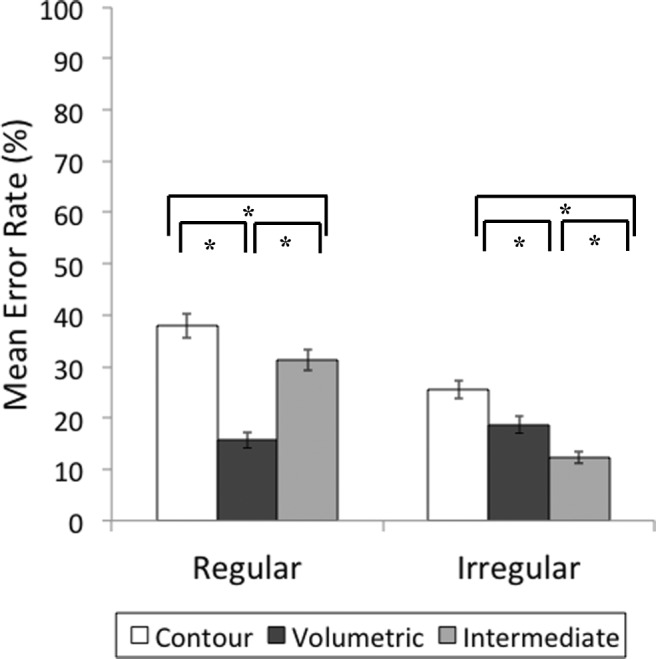
Mean percentage error rates per condition for the match trials in Experiment 1. Error bars show standard error of the mean

Cell means are shown in Fig. 4. The correlation between RT and error rates was significant (*r*^2^ = .4, *p* < .001), suggesting there was no speed–accuracy trade-off.

Nonparametric test was carried out due to the lack of normality in the distribution of errors across conditions. A Friedman test for multiple-dependent groups by ranks was significant, *χ*^2^(11, *N* = 45) = 141.80, *p* < .001. Error rates were further examined using a paired Wilcoxon signed-ranks test. There were more errors for regular (*M* = 28.4, *SD* = 11.17) compared with irregular objects (*M* = 18.7, *SD* = 9.98), *Z* = −4.96, *p* < .001. For regular objects, contour parts yielded more errors than volumetric, *Z* = −4.98, *p* < .001, and intermediate parts, *Z* = −2.37, *p* = .04. Regular volumetric parts yielded fewer errors than intermediate parts, *Z* = −5.02, *p* < .001. Similarly, for irregular objects, there were more errors for contour parts compared to volumetric, *Z* = −2.57, *p* = .04, and intermediate parts, *Z* = −4.33, *p* < .001. However, this time volumetric parts yielded more errors than intermediate parts did, *Z* = −3.03, *p* = .02.

### Discussion

The main findings of Experiment 1 can be summarized as follows. First, the geometric regularity of object shape affected the efficiency of whole–part matching, with better performance for irregular compared with regular objects. Second, parts that contained surfaces, regardless of whether they defined volumetric or nonvolumetric (intermediate) comparison parts, were matched better than closed contour parts that did not form regions corresponding to object surfaces. Third, geometric regularity interacted with part type: volumetric parts were matched better to the whole objects than to intermediate parts for regular objects, but equally well for irregular objects.

Although the current findings speak to the importance of symmetry for the recovery of volumetric structure (e.g., Pizlo et al., 2010; Sawada et al., 2011), the question remains of what is the shape primitive that accounts for the successful matching performance in both regular and irregular objects. One candidate is that volumetric components, derived by the presence of nonaccidental properties in the image, mediate segmentation and representation of regular 3D objects (e.g., Biederman, 1987; Brooks, 1981; Marr & Nishihara, 1978). This type of representation, however, is unsuitable as a general-purpose primitive for objects that cannot be described by regular geometric primitives, such as the geometrically irregular objects used here. Our findings show that image segmentation of irregular objects, was mediated by parts containing surfaces regardless of whether the surfaces were arranged in volumetric (i.e., volume parts) or nonvolumetric configurations (i.e., intermediate parts). To account for the full pattern of results, volumetric models would seemingly need to also allow for the explicit representation of the local pair-wise grouping of edges and vertices into individual bounded regions that make up surfaces. One example of such a model is the JIM3 model (Hummel, 2001), which makes reference to the computation of surfaces, but with no strong theoretical claims about their functional role in the representation. Similarly, although Sawada et al. (2011) propose that once the wireframe contour-based 3D model has been computed it may be “wrapped” in surfaces in order that surface-based attributes (e.g., colour, texture) may be bound to shape to facilitate recognition, computation of surfaces *follows* the computation of a volumetric model of the object. Therefore, even when assuming the computation of surface shape at some point in image processing, accounts that posit the primacy of volumetric structure in image processing of complex objects are unlikely to fully account for the pattern of results in Experiment 1.

An alternative interpretation is in terms of the surface-based model for 3D shape representation proposed by Leek et al. (2005; see also Leek et al., 2016). In the surface-based model, images of complex 3D objects are initially segmented into 2D closed regions, or polygons, approximating visible object surfaces. The configuration of these 2D surface patches is then encoded within a surface configuration map which is used to access a similarly structured long-term memory representation of all known object surfaces. Even though the surface-based model does not contain explicit volumetric part structure as a representational primitive, apparent volumetric grouping effects can arise for *regular* objects as a result of local surface connectivity patterns that can lead to emergent volumetric primitives. That is because, the surface connectivity map encodes pair-wise spatial relationships between adjacent surfaces. The strength of these associations depends, among other factors, on their frequency of co-occurrence across viewpoints. That is, two surfaces that share a common border will develop a strong intercorrelation (i.e., surface connectivity weight). It is these regions of high intercorrelation that are predicted to lead to emergent volumetric structure for groups of spatially adjacent surfaces.

However, such emergent volumetric grouping effects do not appear to arise with *irregular* objects. Why not? One might suppose that similar local surface-adjacency grouping effects should arise regardless of object geometry assuming that surface-based primitives mediate the representation of regular and irregular forms. One explanation may be related to *surface diagnosticity*. Surface diagnosticity refers to how unique a surface is to the object—that is, how frequently it appears across an object set. Recognition of a target object is likely to benefit from presentation of surfaces that are unique to the object—that is, diagnostic surfaces, compared with surfaces shared by other objects. A surface that appears with very low frequency in an object set is more likely to be predictive of a particular target object than a surface that appears often in the object set.

Geometrically regular surfaces are likely to be less unique in predicting object identity (e.g., rectilinear surfaces may appear in several different regular objects). In these circumstances, it may make sense for higher-order local grouping through intercorrelation to be used to constrain the object identification of regular objects (since the addition of further local surfaces will increase the uniqueness of local surface regions). Thus, for regular objects, apparent volumetric effects arising from local surface intercorrelation may occur. In contrast, irregular objects are more likely than regular objects to contain diagnostic surfaces (due to deformations arising from asymmetrical cross-sections, etc.), and therefore are more predictive of object identity. Thus, for irregular objects, local surface intercorrelation may be masked because identification can be based more reliably on individual, highly diagnostic, local surface patches.

Diagnosticity, as a shape-based property, has been reported elsewhere as a criterion for more efficient performance (e.g., Biederman, 1987, p. 131). In particular, more complex (multipart) objects tend to be named faster than simple (single parts) objects, due to redundancy gain from other possible matches, affording them higher discriminability in memory. For surface-based models, diagnosticity is the property of the shape of 2D edge-bounded regions that correspond to object surfaces.

To examine this account of our results, surface diagnosticity was calculated for each object—that is, how often a surface was likely to occur in the entire object set. The diagnostic value of each surface in the current object set was determined in terms of a number of nonaccidental and metric properties (see also Witkin & Tenenbaum, 1983, for a similar way to quantify the uniqueness of apparent surface quality). Each surface was described in terms of four categorical nonaccidental properties: symmetry (symmetrical vs. asymmetrical), parallelism (parallel vs. converging), straightness of axis (straight vs. curved), straightness of cross-section (straight vs. curved). Each surface was also described in terms of three metric properties: aspect ratio (elongated vs. equilateral), number of axes of symmetry (one or two), and number of edges (four to seven). These dimensions gave rise to 10 different surface shapes (parallelogram, ellipse, triangle, trapezoid, trapezium, rhombus, skewed rhombus, pentagon, hexagon, and heptagon). Diagnosticity was calculated as the inverse probability value of occurrence of that surface in the entire set of surfaces. Surfaces from irregular objects (*M* = .95, *SD* = .06) were more diagnostic than surfaces from regular objects (*M* = .85, *SD* = .09), *t*(6) = −5.19, *p* < .001.

A Pearson correlation showed that the combined surface diagnosticity of volumetric and surface comparison parts correlated negatively with response latencies—higher diagnosticity values were correlated with faster RT, *r*^2^ = .34, *p* = .003. This result lends support to the proposal that the high diagnosticity of individual object surfaces in irregular objects may have led to fast RT regardless of whether the surfaces formed a volume (as in the case of volumetric parts) or not (as in the case of intermediate parts).

## Experiment 2

Experiment 2 was motivated by four independent objectives. First, and most importantly, Experiment 2 examined whether surface diagnosticity can account for the Regularity × Part Type interaction found in Experiment 1. Surface diagnosticity was manipulated here by creating novel objects, which had either none (high diagnosticity) or more than one (low diagnosticity) surfaces in common with other objects. Second, a recognition memory task was used, rather than whole–part matching, to more directly explore shape representations mediating recognition as opposed to perception. Third, a priming task was used to obtain an implicit measure of the nature of shape representations. The predictions of volumetric and surface-based models were contrasted. Volumetric accounts, where 3D object structure can be computed from 2D edge-based information, such as nonaccidental properties, would not predict an influence of surface diagnosticity on primed object recognition (e.g., Biederman, 1987; Brooks, 1981; Marr & Nishihara, 1978). According to volumetric part-based accounts, all surfaces are equally predictive of object identity—not by virtue of being surfaces (as models based on the approximation of shape primitives from nonaccidental properties do not necessarily assume a functional role for surfaces) but by virtue of containing sufficient edge-based nonaccidental properties to give rise to a volumetric structural description. The nonaccidental information present in both the volumetric and intermediate parts would be sufficient to aid recovery of the complete corresponding object volumes irrespective of object shape regularity. Similarly, accounts, where 3D structure can be computed from 2D edge-based descriptions of objects following global shape constraints, such as symmetry (e.g., Li et al., 2013; Pizlo et al., 2010; Sawada et al., 2011), would not predict the influence of surface diagnosticity on primed recognition, especially for regularly shaped objects.

Meanwhile, surface-based accounts (e.g., Leek et al., 2005; Leek et al., 2009; Leek et al., 2016) would predict a significant effect of surface diagnosticity, a local shape property of areas on the image corresponding to object surfaces, and its significant interaction with prime type: for low diagnosticity objects, volumetric primes would yield better recognition compared with nonvolumetric (intermediate) primes, due to learned intercorrelations between adjacent surfaces of each volume. However, for high surface diagnosticity objects, such intercorrelations would be superseded by the influence of surface diagnosticity, with no difference in primed recognition between volumetric and nonvolumetric primes.

### Method

#### Participants

Forty students and staff at Swansea University Psychology department (*M*_age_ = 28 years, *SD* = 8.0) participated either voluntarily or in exchange for course credit. Participants were randomly allocated to either the volumetric primes group (*N* = 20) or the intermediate primes group (*N* = 20) at the beginning of the experiment. They all reported normal or corrected-to-normal vision.

Having each participant exposed to either volumetric or nonvolumetric (intermediate) configuration of surfaces was necessary in order to keep the number of trials manageable for each participant within a single experimental session. Note that because surface diagnosticity was examined in Experiment 2, it was important that each participant was exposed to the entire stimulus set during the learning phase, thus making it possible to acquire information over time about the relative diagnosticity value of each surface.

#### Apparatus

Trial presentation and recording of responses was controlled via PsyScope (Cohen, MacWhinney, Flatt, & Provost, 1993). Stimuli were displayed on an Apple Macintosh G4 computer via a 17-inch RGB monitor, at a viewing distance of approximately 60 cm.

#### Stimuli

**Fig. 5:**
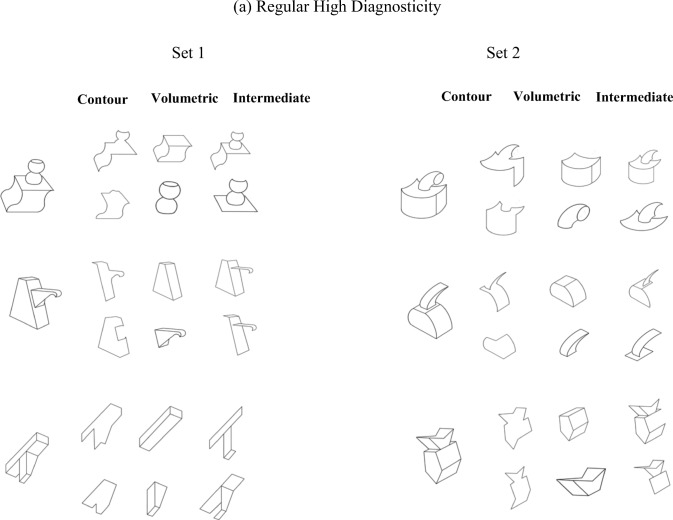

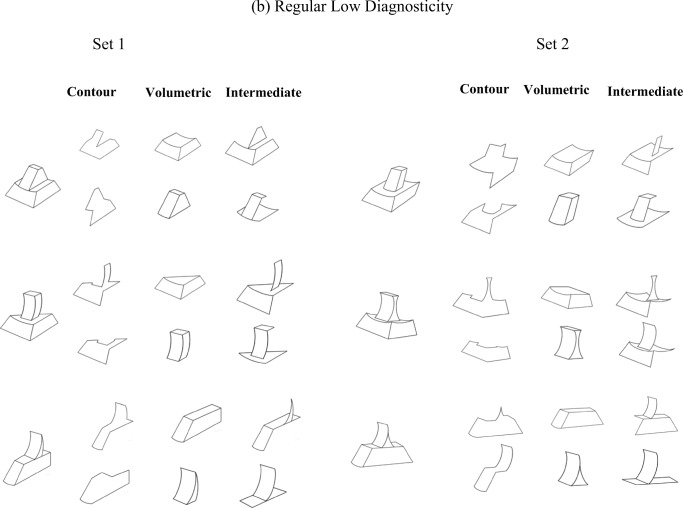

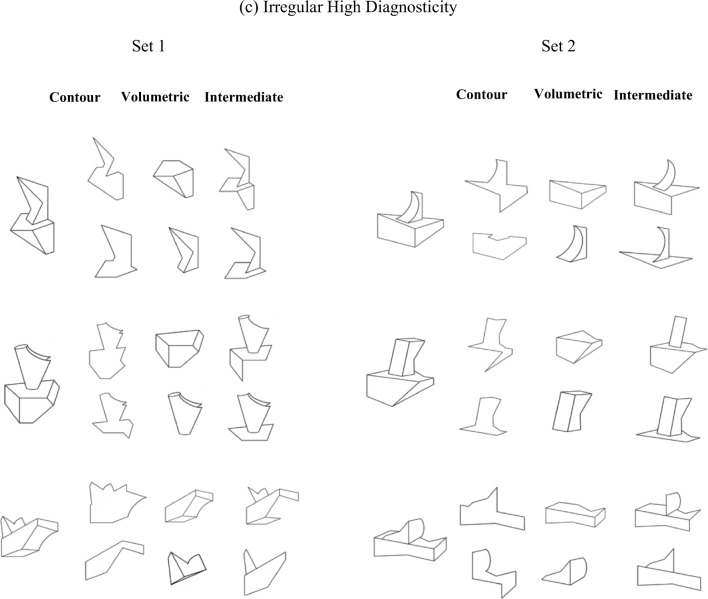

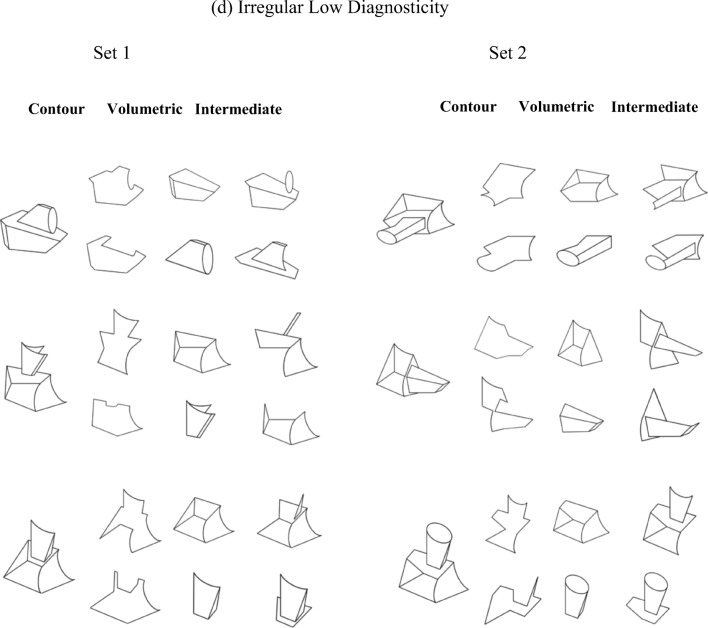
Twenty-four objects and their associated primes used in Experiment 2. Half of participants studied Set 1 objects; the other half studied Set 2 objects. Object related primes were one of the primes of that object (contour and volumetric for the volumetric primes group; contour and intermediate for the intermediate primes group). Unrelated primes were the primes of the object opposite it in the figure (e.g., unrelated primes for the first object of Set 1 were the related primes of the first object in Set 2, and vice versa). All participants saw the contour primes. Participants in the volumetric group also saw volumetric primes, and participants in the intermediate primes group also saw intermediate (or surface) primes (i.e., primes consisting of some of the object’s surfaces arranged in a nonvolumetric configuration)

Twenty-four opaque black-and-white line drawings of novel three-dimensional objects were used. Each were fitted within a 6 × 6 cm frame (not visible during the experiment) subtending 6.2^o^ (see Fig. 5). The stimuli were created by hand using Adobe Photoshop. Every effort was made to avoid creating objects that might look like familiar objects. Below are the constraints followed for creating the stimuli in Experiment 2.

#### Two-component structure

Each whole object consisted of two volumetric components at a clearly defined region of paired concave minima of curvature (Biederman, 1987; Hoffman & Richards, 1984). The two volumetric components were defined by variation of the following parameters: curvature of the main axis, tapering (parallelism), edges (straight vs. curved), and symmetry of the cross section (Biederman, 1987). Visual similarity among components in the object set varied according to changes in these parameters. Each volumetric part could be uniquely specified by a combination of nonaccidental property (NAP) relations and aspect ratio. All stimuli shared the same spatial configuration, where an “end-on” relation attaches one component to the other. This ensured that discrimination among stimuli required attention to the shapes of the individual components. The stimuli were depicted from a single three-quarter view- point that was chosen to maximize visibility of object structure. Twelve naïve participants in a pilot study confirmed the two-component structure of each stimulus. There was 100% agreement about the number of volumetric parts and about the location of the volumetric part boundary.

##### Regularity

Following the same general principles as Experiment 1, 12 of the 24 objects were composed of two geometrically *regular* components and the remaining 12 objects were composed of two geometrically *irregular* components.

##### Surface diagnosticity

The diagnostic value of each surface in the current object set was determined in terms of the number of times a surface appeared in a set of objects. Surface diagnosticity was calculated separately for regular and irregular objects. A surface was considered to be low in surface diagnosticity if it appeared more than once in the set of objects. Surfaces in the regular and the irregular object set were counted, and an object was assigned a diagnosticity value depending on how may reoccurring surfaces it contained and how many times each surface appeared in the set. For example, in the regular set of objects there were 68 visible and partially visible surfaces in those objects. The top-left object in Fig. 5b (regular low diagnosticity) contains three surfaces that reappear in the remaining five regular, low diagnosticity objects. Two of those three surfaces appear twice, and the other appears four times. Therefore, the diagnosticity value of that object is calculated as follows: [(4 + 2+2) × 100]/68 = 11.76. In terms of inverse probability, the surface diagnosticity value is 100 minus 11.76 = 88.24, or .88.

Low diagnosticity objects had a mean surface diagnosticity value of (*M* = .84, *SD* = .30), while high diagnosticity objects had a diagnosticity value of (*M* = 1.00, *SD* = .00). Low diagnosticity irregular objects had a surface diagnosticity value of (*M* = .85, *SD* = .37) and low diagnosticity regular objects had a surface diagnosticity value of (*M* = .84, *SD* = .21). Low diagnosticity objects differed significantly from high diagnosticity objects both for regular and for irregular objects, *t*(10) = 12.87, *p* < .001, and *t*(10) = 9.77, *p* < .001, respectively.

##### Three prime types

For each object, three types of prime stimuli were created: *closed contour*, *volumetric*, and *intermediate* primes. The volumetric surface configuration primes consisted of one of the two components of the object, while the intermediate primes consisted of the same number of surfaces as the volumetric configuration parts ensuring that the surfaces did not form a volume. The closed contour component parts were made by deleting regions of object contour with the constraint that the resulting image was a closed form that did not correspond to any complete object surface. To prevent contour overlap between the whole object and the comparison parts, the whole object stimuli were enlarged by 150% of their original size.

Surface diagnosticity was calculated as above for each prime type. Specifically, the mean surface diagnosticity value for a volumetric prime was calculated based on how many times each of the surfaces on the prime appear in the other volumetric primes in the object set. The same principle applied for intermediate primes. The mean surface diagnosticity values per prime type (volumetric vs. intermediate) appear in Table 2. Low diagnosticity primes differed significantly from high diagnosticity primes both for volumetric and intermediate primes, *t*(10) = 12.25, *p* < .001, and *t*(10) = 16.36, *p* < .001, respectively.

**Table 2:** Summary of low-level properties of the contour, volumetric, and surface primes for the 12 low and 12 high surface diagnosticity objects, used in Experiment 2

	Prime Type	Edge contour (%)	Bounding contour (%)	*N* vertices				Surface diagnosticity value
				L	Y	T	% vertex change	
		Mean (*SD*)	Mean (*SD*)	Mean (*SD*)	Mean (*SD*)	Mean (*SD*)	Mean (*SD*)	Mean (*SD*)
Surface diagnosticity								
Low	Contour	40.47 (5.09)	26.79 (4.34)	9.42 (1.16)	N/A	N/A	78.02 (12.78)	N/A
	Volumetric	42.33 (3.01)	28.24 (3.86)	2.62 (0.53)	3.08 (0.90)	1.96 (0.65)	10.88 (13.21)	.839 (.31)
	Intermediate	48.91 (6.78)	33.04 (5.68)	6.37 (0.88)	2.29 (0.40)	2.71 (0.50)	51.84 (7.33)	.852 (.30)
High	Contour	46.77 (3.45)	26.63 (3.12)	10.08 (1.63)	N/A	N/A	73.82 (8.44)	N/A
	Volumetric	45.42 (7.89)	27.42 (3.93)	3.33 (0.83)	3.25 (0.81)	2.04 (0.91)	10.33 (11.05)	.997 (.00)
	Intermediate	49.61 (5.66)	34.31 (4.43)	7.58 (0.87)	1.96 (0.69)	2.50 (0.48)	(48.95 (7.15)	.997 (.00)

In the N/A cells there are no corresponding values because the area enclosed in the contour primes did not correspond to any of the objects’ surfaces

#### Summary of low-level features

Table 2 shows low-level image properties for each comparison part.

#### Bounding contour

There was no overall difference in bounding contour between high and low surface diagnosticity objects, *t*(22) = 0.67, *p* > .05.

For low diagnosticity objects, intermediate parts contained the most bounding contour compared with both volumetric, *t*(11) = 6.69, *p* < .001, and contour parts, *t*(11) = 7.23, *p* < .001, while volumetric parts contained more bounding contour than contour parts, *t*(11) = 2.61, *p* = .02. For high diagnosticity objects, intermediate parts contained more bounding contour than both volumetric, *t*(11) = 10.26, *p* = .002, and contour parts, *t*(11) = 9.55, *p* = .002, while there was no difference in bounding contour between contour and volumetric parts, *t*(11) = 1.06, *p* > .05.

#### Vertices

##### Low versus high diagnosticity

Objects with high surface diagnosticity contained significantly more L vertices than did objects with low surface diagnosticity, *t*(22) = 2.48, *p* = .02, *p* = .007, while there was no significant difference between objects with high and low surface diagnosticity in terms of Y vertices, *t*(22) = 0.48, *p* > .05; T vertices, *t*(22) = 0.31, *p* > .05; or vertex change, *t*(22) = 1.08, *p* > .05.

For low diagnosticity objects: contour primes contained significantly more L vertices than volumetric and intermediate primes, *t*(11) = 19.12, *p* < .001, and *t*(11) = 9.75, *p* < .001, respectively. Intermediate primes contained significantly more L vertices, *t*(11) = 13.14, *p* > .001, and more T vertices than volumetric primes, *t*(11) = 4.78, *p* < .001, and significantly fewer Y vertices than volumetric primes, *t*(11) = 2.37, *p* = .04. The percentage of vertex change was significantly higher for contour primes compared with both volumetric primes, *t*(11) = 11.52, *p* < .001, and intermediate primes, *t*(11) = 8.34, *p* < .001. Intermediate primes had a significantly higher proportion of vertex change than volumetric primes, *t*(11) = 8.76, *p* < .001.

For high diagnosticity objects, contour primes contained significantly more L vertices than did volumetric and intermediate primes, *t*(11) = 13.76, *p* < .001, and *t*(11) = 5.92, *p* < .001, respectively. Intermediate primes contained significantly more L vertices than did volumetric primes, *t*(11) = 15.64, *p* < .001, significantly fewer Y vertices than volumetric primes, *t*(11) = 8.26, *p* < .001, while there was no difference in terms of T vertices, *t*(11) = 1.45, *p* > .05. The percentage of vertex change (e.g., from T or Y to L) was significantly higher for contour primes compared with both volumetric primes, *t*(11) = 15.94, *p* < .001, and intermediate primes, *t*(11) = 7.66, *p* < .001. Intermediate primes had a significantly higher level of vertex change than volumetric primes did, *t*(11) = 9.28, *p* < .001.

#### Surface diagnosticity manipulation check

The objects in Experiment 2 were designed such that 12 objects had no surfaces shared with any other object, while the remaining 12 objects shared one or more surfaces with other objects in their set—*high* and *low* surface diagnostic objects. Nevertheless, surface diagnosticity values were computed for each object and are shown in Tables 2 and 3. Objects with high surface diagnosticity had lower inverse probability of a surface occurring than did low surface diagnosticity objects, *t*(11) = 22*.46*, *p* < .001.

**Table 3:** Mean RTs (and standard deviations in parentheses) and priming effects per surface diagnosticity, relatedness, prime type, and group in Experiment 2

	Diagnosticity	Related		Unrelated		No prime	
		Low	High	Low	High	Low	High
		Mean RT (*SD)*	Mean RT (*SD*)	Mean RT (*SD*)	Mean RT (*SD*)	Mean RT (*SD*)	Mean RT (*SD*)
Intermediate primes group	Contours	1,004.29 (250.02)	859.30 (181.37)	1,067.76 (329.44)	892.95 (194.41)	979.09 (207.14)	923.80 (225.50)
	Intermediate	1,003.58 (291.88)	838.32 (188.42)	974.23 (205.30)	920.96 (190.42)		
Volumetric primes group	Contours	913.13 (242.17)	821.77 (203.92)	950.78 (268.20)	790.09 (157.31)	976.38 (204.45)	805.32 (131.24)
	Volumes	848.26 (154.85)	806.24 (192.96)	986.79 (277.24)	801.61 (181.80)		

##### Image similarity

Two measures of image similarity were computed for the high and low diagnosticity conditions: (1) image pixel intensity (normalised sums of squared differences in pixel intensity); (2) HMAX C1 output layer (Serre, Oliva, et al., 2007) similarity values. These measures were computed using the MATLAB Image Similarity Toolbox (Seibert & Leeds, https://github.com/daseibert/image_similarity_toolbox), which outputs the similarity measures for each image (within each respective stimulus type condition). The C1 layer outputs were used to approximate a measure of image similarity that incorporates scale and position invariance. These values were then normalised between 0 and 1 relative to the maximum similarity score across all conditions with higher values indicating greater image similarity.

There was no significant difference in mean normalised pixel intensity for high (*M* = 0.59, *SD* = .06) and low (*M* = 0.59, *SD* = .08) diagnosticity objects.

HMAX mean normalised similarity values were higher in the low (*M* = 0.47, *SD* = .06) than in the high (*M* = 0.45, *SD* = .05) diagnosticity condition, *t*(166) = 2.44, *p* = .01.

#### Design

The study was based on a mixed design with target response (yes vs. no), prime relatedness (related vs. unrelated), object regularity (regular vs. irregular), surface diagnosticity (high vs. low), and prime type (contour, surface-based, and no prime) as the within-participants factors. The between-participants factor was group with two levels: volumetric components versus intermediate primes. In the *volumetric primes* group, objects were primed either with one of the two volumetric components from each object (volumetric primes) or with regions of closed contour that did not correspond to any object surfaces (contour primes). In the *intermediate primes* group, objects were primed with a number of adjacent surfaces that did not make up a volume (intermediate primes), or with regions of closed contour that did not correspond to any of the object surfaces (contour primes). The contour primes were the same for both groups. The number of surfaces contained in the surface primes in the *intermediate primes* group were the same as the number of surfaces in the volumetric primes in the *volumetric primes* group.

Each participant in each group saw 480 trials, which consisted of 12 trials per experimental condition, and a total of 96 no-prime trials, where only a mask preceded the object. The dependent measure was response times (RTs).

#### Procedure

There were three phases: the copy-draw phase, the computerised learning phase, and the computerised test phase. In the copy-draw phase, participants were shown 12 objects to copy-draw on a separate piece of paper. Half of the participants learned one set of 12 objects and the other half learned a different set of 12 objects (see Fig. 5). For participants who learned Set 1 objects, the primes in that set were the related primes while the primes in Set 2 were the unrelated primes. For a participant who learned the objects from Set 1, the object in the top-left corner of Fig. 5a would be preceded either by one of its own prime stimuli (related primes) or by primes from the object directly opposite it on the right side of Fig. 5 (unrelated primes). This was reversed for participants who learned the Set 2 objects. Participants were given unlimited time to complete the learning phase, although approximately 20 minutes was typically required.

In the computerised learning phase, each trial began with the presentation of a fixation cross in the centre of the screen for 750 ms. There was then an interstimulus interval (ISI) for 750 ms, followed by the presentation of an object for 5 seconds or until a response was given. The participant was required to indicate whether the object was previously learned (during copy-drawing) by pressing *Z* on the keyboard to indicate yes, or *M* to indicate no. The distracter objects for participants who studied Set 1 objects were the target objects for participants who studied Set 2 objects, and vice versa. Each target and distractor object appeared in random order three times, yielding 72 trials in this phase of the experiment, and each participant was required to achieve approximately 90% accuracy to ensure that they had successfully learned the target objects. Errors were followed by auditory feedback in the form of a beep. If more than five errors were made then the experimenter repeated the computerised learning phase until the participant achieved the required standard. If a rerun were necessary, then the participant was asked to have another look at the target objects (without copy-drawing) before the computerised learning phase was repeated.

The test phase began after successful completion of the learning phase. Each trial began with a fixation cross for 750 ms, followed by an ISI for 400 ms. A prime (closed contour primes, volumetric, or a blank screen, for the volumetric primes group; contour primes, intermediate primes, or a blank screen in the intermediate primes group) then appeared for 250 ms, followed by another ISI for 150 ms, and then a mask for 250 ms. Another ISI lasting 250 ms was then followed by the presentation of a whole object until a response was given. Participants indicated whether or not the object was one of those previously learned. There were 480 trials with an opportunity to take a break halfway.

#### Data analysis

##### Response times

For the response times (RT) analyses, only data from the yes trials—that is, trials where the object was one of those studied during the learning phase—were analysed. Correct mean RT is analysed separately, in terms of object shape regularity and in terms of surface diagnosticity.

##### Priming effects

One set of priming effects was calculated using the RT from the no-prime trials (no-prime RT minus related RT per prime type, regularity, and diagnosticity). Another set of priming effects was calculated using the RT from the unrelated primes (unrelated RT minus related RT per prime type, regularity, and diagnosticity). Priming effects showed the same pattern of results and significance, regardless of how they were calculated, so only priming effects calculated using the no-prime trials RT are reported.

### Results

#### Error rates

Errors (*M* = 9.05%, *SE* = 1.3%) were removed from the data and not analysed further. Correct response times (RT) were trimmed to ±2 standard deviations from the mean per condition, which led to the removal of 2.1% from the total number of trials.

#### Response times

**Fig. 6:**
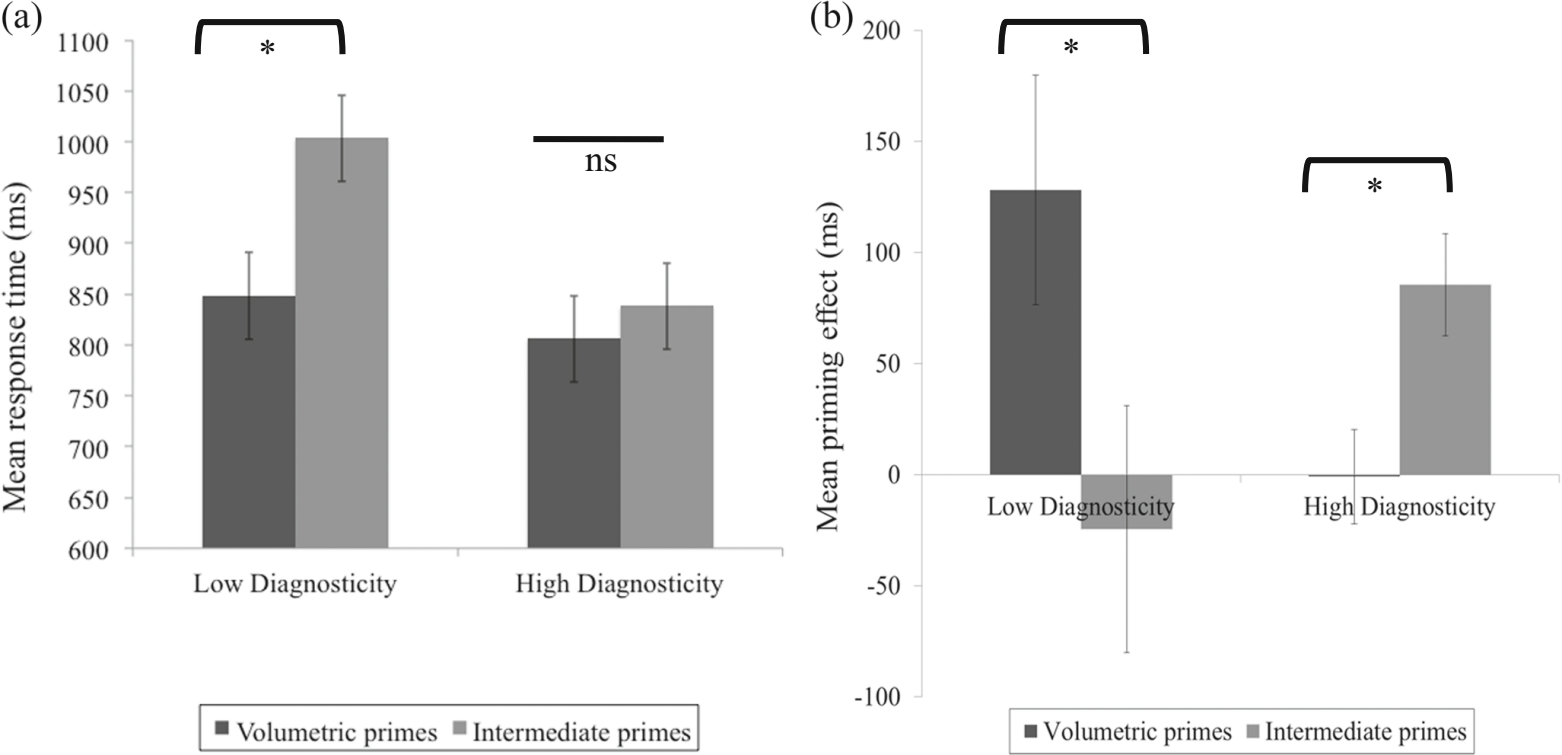
**a** Mean response times (milliseconds) per condition for objects with low and high surface diagnosticity in Experiment 2. **b** Mean priming effects (using the no prime) per condition (low vs. high surface diagnosticity) and per group (volumetric primes vs. intermediate primes). Error bars show standard error of the mean

Analysis of RTs was carried out on correct responses. Cell means for the key conditions are shown in Fig. 6.

Mean RTs were faster for target trials (yes response, *M* = 826.97, *SE* = 28.28) than for nontarget trials (no response, *M* = 922.74, *SE* = 23.94), *t*(39) = 5.22, *p* < .001. The remaining analyses were restricted to target present (yes response) trials.

Table 3 shows the mean RT and priming effects for target-present trials (yes trials) as a function of object surface diagnosticity, group (volumetric vs. intermediate), prime relatedness (related vs. unrelated), and prime type (contour based vs. surface based).

A 2 (relatedness: related, unrelated) × 2 (regularity: regular vs. irregular) × 2 (diagnosticity: high vs. low) × 2 (group: volumetric vs. intermediate primes) × 2 (prime type: contour based vs. surface based) mixed ANOVA, with group as the between-participants factor, revealed a significant main effect of relatedness, *F*(1, 38) = 9.56, *p* = .04, with related RT faster than unrelated RT. There was also a significant four-way interaction involving relatedness, regularity, diagnosticity and group, *F*(1, 38) = 4.17, *p* = .02, η_p_^2^ =.10. Given the significant main effect of relatedness, and its involvement in a four-way interaction, the analysis was concentrated only on *related* trials in order keep the results section concise and directly relevant to the aims of Experiment 2. These were the trials where the prime was from the object succeeding it.

A 2 (regularity: regular vs. irregular) × 2 (diagnosticity: high vs. low surface diagnosticity) × 2 (prime type: contour-based vs. surface-based) × group (volumetric vs. intermediate) on related trial RT showed a significant three-way interaction involving diagnosticity, prime type, and group, *F*(1, 38) = 4.24, *p* = .05, η_p_^2^ =.10.

To examine the interaction, separate analyses were carried out on contour-based and surface-based primes. A 2 (regularity: regular vs. irregular) × 2 (diagnosticity: high, low) × 2 (group: volumetric vs. intermediate surface configuration) mixed ANOVA on correct RT for trials with *contour-based primes*, showed a significant main effect of diagnosticity, *F*(1, 38) = 19.40, *p* < .001, η_p_^2^ =.338, with slower RT for low compared with high diagnosticity objects, and a significant main effect of regularity, *F*(1, 38) = 11.52, *p* = .002, η_p_^2^ =.233, with slower RT for irregular compared with regular objects. The Regularity × Diagnosticity interaction was significant, *F*(1, 38) = 16.94, *p* < .001, η_p_^2^ =.308. For irregular objects, surface diagnosticity did not make a difference in RT, *t*(39) = .058, *p* > .05. However, regular objects with high diagnosticity (*M* = 743.68, *SE* = 28.17) yielded faster RT than their low diagnosticity counterparts (*M* = 966.26, *SE* = 48.11), *t*(39) = 6.07, *p* < .001. There were no other significant effects. The lack of a significant main effect or interaction involving group (volumetric vs. intermediate) suggests that RT for contour-based primes (which were the same for the volumetric primes and the intermediate primes groups) was similar for the group seeing the volumetric primes interspersed with the contour primes, to the group who saw intermediate primes interspersed with the same contour primes.

A different 2 (regularity: regular vs. irregular) × 2 (diagnosticity: high, low) × 2 (group: volumetric vs. intermediate surface configuration) mixed ANOVA, on correct RT from related *surface-based prime* trials only, showed a significant main effect of regularity, *F*(1, 38) = 36.39, *p* < .001, η_p_^2^ = .489, with slower irregular RT compared with regular RT, and a significant main effect of diagnosticity, *F*(1, 38) = 26.33, *p* < .001, η_p_^2^ =.409, with slower RT for low compared with high surface diagnosticity objects. The Regularity × Diagnosticity interaction was significant, *F*(1, 38) = 12.45, *p* < .001, η_p_^2^ = .247, but regularity was not involved in any other interactions. Critically, there was a significant Diagnosticity × Group interaction, *F*(1, 38) = 6.87, *p* < .01, η_p_^2^ =.153. For low diagnosticity objects, *volumetric* primes (*M* = 873.71, *SD* = 196.67) led to faster RTs than did *intermediate* primes (*M* = 1038.09, *SD* = 281.76), *t*(38) = 2.14, *p* = .04. In contrast, for high diagnosticity objects, there was no difference between *volumetric* (*M* = 802.63, *SD* = 182.07) and *intermediate* primes (*M* = 835.98, *SD* = 189.92), *t*(38) = .57, *p* > .05. In summary, low diagnosticity objects were recognised faster if surfaces were in a volumetric configuration (volumetric primes) compared with when surfaces were arranged in a nonvolumetric configuration (intermediate primes). For high diagnosticity objects, however, there was no such difference in recognition latencies.

#### Priming effects

Priming effects are shown in Fig. 6. Priming effects were calculated using both the unrelated prime RT and the no-prime trial RT. As they showed the same pattern and significance of priming across conditions, only priming effects calculated using the no-prime trial RT were reported (see Table 3 and Fig. 6).

A 2 (regularity: regular vs. irregular) × 2 (diagnosticity: high, low) × 2 (group: volumetric vs. intermediate) × 2 (prime type: contour vs. surface-based primes) mixed ANOVA on priming effects, with group as the between-participants factor, only showed a significant three-way interaction involving diagnosticity, prime type, and group, *F* (1, 38) = 4.24, *p* = .04, η_p_^2^ =.10. The interaction was examined by carrying out separate analyses for contour-based and surface-based primes.

A 2 (diagnosticity: high, low) × 2 (group: volumetric vs. intermediate) mixed ANOVA on priming effects for the *contour-based* primes for each group revealed no significant effects or interactions. The same ANOVA on priming effects for *surface-based primes* revealed a significant interaction, *F*(1, 38) = 11.31, *p* = .002, η_p_^2^ =.23. Low diagnosticity objects were primed better by volumetric than by intermediate primes, *t*(38) = 2.98, *p* = .005. The opposite was true for high diagnosticity objects, where there was better priming for intermediate primes compared with volumetric primes, *t*(38) = 2.36, *p* = .02.

Therefore, the pattern of priming approximately mirrored the pattern of RTs: Low diagnosticity objects were recognised faster if they were primed by volumetric compared with intermediate primes, while the opposite was true for high diagnosticity objects.

#### Analyses of significant image properties on RTs

For each of the dimensions in which image properties differed significantly, tests were performed to determine the strength of the relationship between the image dimension and observed RT.

High and low surface diagnosticity objects differed in the inverse probability of a surface appearing—there was a higher probability of a surface appearing more than once in low surface diagnosticity objects. A correlation between related trial RT and surface diagnosticity value was significant, *r*^2^ < .478, *p* < .001, confirming the relationship between recognition times and diagnosticity of the surfaces appearing in the prime stimulus.

Surface primes contained more *bounding contour* than volumetric and contour parts, for both high and low diagnosticity objects. There was no significant correlation between related trials RT and bounding edge contour for three primes types in either the high (*r*^2^ = .18, *p* > .05) or the low diagnosticity objects (*r*^2^ = .17, *p* > .05).

Volumetric primes contained significantly fewer L vertices and significantly more Y vertices than intermediate primes for both high and low surface diagnosticity objects. Nevertheless, the correlation between L and Y vertices and RTs was not significant for either high (*r*^2^ < .34, *p* > .05, and *r*^2^ < .13, *p* > .05 for L and Y vertices respectively) or for low surface diagnosticity objects (*r*^2^ < .38, *p* > .05, and *r*^2^ < .05, *p* > .05 for L and Y vertices, respectively). For low diagnosticity objects, intermediate primes contained more T vertices than volumetric primes, but the correlation between number of vertices and mean RT was not significant (*r*^2^ < .22, *p* > .05). Finally, intermediate primes had a larger proportion of vertex change than volumetric primes for both high and low diagnosticity objects, but the correlations with mean RT was not significant (*r*^2^ < .19, *p* > .05 and *r*^2^ < .27, *p* > .05, respectively). These results suggest the pattern of differences in RT and in the priming effects cannot be accounted for by low-level image differences.

### Discussion

For surface-based models, diagnosticity is the property of the shape of 2D edge-bounded regions that correspond to object surfaces. Experiment 2 examined whether surface diagnosticity—that is, the uniqueness of a surface across an object set—contributed to the pattern of results in Experiment 1, namely, the apparent volumetric benefit for regular objects but not for irregular objects. Specifically, in Experiment 1, it was hypothesized that geometrically regular surfaces (e.g., rectilinear surfaces may appear in several different regular objects) were likely to be less unique in predicting object identity. In these circumstances, it may make sense for higher-order local grouping through intercorrelation to be used to constrain the object identification of regular objects (since the addition of further local surfaces will increase the uniqueness of local surface regions). Thus, for regular objects, apparent volumetric effects arising from local surface intercorrelation may occur as regular objects contain low diagnostic surfaces. For irregular objects, effects of the representation of local surface intercorrelation may be masked because identification can be based more reliably on individual, highly diagnostic local surface patches.

In Experiment 2, surface diagnosticity was manipulated by creating novel objects, which had either none (high diagnosticity) or one or more (low diagnosticity) surfaces in common with other objects in the studied set. In order to separate effects of shape regularity from effects of surface diagnosticity, a novel object set was created were shape regularity and surface diagnosticity were orthogonally controlled. The results showed that regardless of whether objects were regular or irregular, objects containing surfaces with low surface diagnosticity were recognised *faster* following volumetric primes than intermediate primes. In contrast, objects with high surface diagnosticity were recognised equally fast regardless of whether the prime was volumetric or nonvolumetric (intermediate). In terms of recognition priming, the pattern of results is similar to that shown in RT.

The results of Experiment 2 show that irrespective of object shape regularity, objects with low diagnostic surfaces benefitted from volumetric configurations of surfaces—a local interconnectivity benefit. In contrast, when surface diagnosticity was high, there was no difference in recognition RT between intermediate and volumetric primes. The finding that surfaces are used as image primitive for object recognition performance regardless of global shape attributes, such as shape regularity, is inconsistent with accounts where 3D structure can be computed from 2D edge-based descriptions of objects following global shape constraints, such as symmetry—an important concept in shape regularity (e.g., Li et al., 2013; Pizlo et al., 2010; Sawada et al., 2011).

The current findings are also inconsistent with the predictions of structural description accounts where 3D object structure can be computed from 2D edge-based information, such as nonaccidental properties. According to some volumetric accounts (e.g., Brooks, 1981; Marr & Nishihara, 1978), all object surfaces are equally predictive of object identity, and the nonaccidental information present in both the volumetric and intermediate parts would be sufficient to aid recovery of the complete corresponding object volumes irrespective of object shape regularity. Yet in Experiment 2, nonaccidental properties present in low-level image features (e.g., L, Y, and T vertices) did not predict the pattern of RT or error performance.

## General discussion

Two experiments examined the shape primitives that may be computed and used to represent complex object shape. In Experiment 1, whole–part matching performance was better for irregular than for regular component parts in both RTs and errors. Furthermore, regularity interacted with part type: For regular objects, *volumetric parts yielded better* performance than nonvolumetric (intermediate) parts did, while for *irregular* objects there was *no difference* in performance between surface and volumetric parts.

In Experiment 2, where surface diagnosticity and object shape regularity were separately manipulated, object shape regularity no longer interacted with the different kinds of shape primitive. Instead, it was surface diagnosticity—a measure of how unique a surface is across the studied object set—that influenced performance *even for regular* objects, where a volumetric image primitive could more readily be computed based on symmetry and simplicity constraints (e.g., Pizlo et al., 2010; Sawada et al., 2011). Specifically, objects with low surface diagnosticity were better primed by volumetric primes, while objects with high surface diagnosticity showed the opposite pattern of results. It is relevant to note that this interaction rules out a possible account of the results based solely on differences in HMAX similarity measures between object sets (see Method section). Notably, low diagnosticity objects were found to have higher HMAX C1 output scores, suggesting greater similarity (and potentially lower discriminability) relative to high diagnosticity objects. While this is expected due to higher similarity (lower diagnosticity), it cannot account for the contrasting patterns of priming found for volumetric and intermediate/surface primes. The current findings cannot be fully explained by hierarchical, feedforward, deep networks (e.g., Kheradpisheh et al., 2016; Krizhevsky et al., 2012; LeCun et al., 2015; Riesenhuber & Poggio, 1999; Serre, Oliva, et al., 2007). These networks compute shape representations using multidimensional feature descriptors. However, they make no explicit reference to the representation, or derivation, of higher-order shape geometry, such as surfaces or volumes (see also Ullman, Assif, Fetaya, & Harari, 2016). Any adequate theory of 3D object shape representation for human vision needs to have the adaptive flexibility to encode, compute, and classify both geometrically regular and irregular object shape and support several different kinds of tasks (e.g., image classification, reaching and grasping). An image primitive based on regularized approximations to volumetric object parts (e.g., Biederman, 1987; Marr & Nishihara, 1978), or one based solely on hierarchical groupings of edge-based fragments (e.g., Edelman, 1999; Edelman & Intrator, 2000; Tarr & Bulthoff, 1995; Ullman & Basri, 1991), is insufficient for this purpose.

A more parsimonious account for the current findings is the hypothesis that object recognition is mediated by *surface-based descriptions* of object shape (e.g., Leek et al., 2005). According to this hypothesis, edge-based descriptions of 3D objects are used to define constituent surfaces and the surface-based description is used to access, or index, stored shape representations during recognition (see also Fan, Medioni, & Nevatia, 1989; Fazl, Grossberg, & Mingolla, 2009; Fisher, 1989; Phillips, Todd, Koenderink, & Kappers, 2003). On the original hypothesis outlined by Leek et al. (2005), shape indexing is achieved by approximating surface shape and accessing stored object representations based on pair-wise spatial configurations of spatially adjacent surfaces. Thus, recognition is based on local surface configuration and does not require the derivation of global object attributes (e.g., principal axis elongation, symmetry). According to this hypothesis, both regular and irregular objects can be represented in terms of surface-based descriptions. For regular objects, apparent volumetric effects arising from local surface intercorrelation may occur as regular objects contain mainly low diagnostic surfaces. For irregular objects, effects on recognition from local surface intercorrelation may be masked because recognition can be based on individual, highly diagnostic, local surface shape.

The hypothesis that surface-based image primitives are used to describe object shape in long-term memory is not necessarily incompatible with recent demonstrations from computational modelling supporting the use of edge-based (rather than surface-based) reconstructions of 3D object geometry in human vision (e.g., Pizlo et al., 2010; Sawada et al., 2011). That is because the hypothesis does not assume, nor require, that surfaces are computed *directly* from perceptual input. For example, Pizlo and colleagues have elegantly shown how veridical 3D structure can be reliably computed during perception from 2D edge-based descriptions of objects following simplicity constraints (e.g., symmetry, complexity). This is accomplished without inferring object surface structure directly from perceptual input, but instead is based on the recovery of a 3D “wireframe” shape description. Note, however, that the recovery of 3D shape from the image and the representation of 3D object shape (i.e., creating a 3D perceptual representation and matching it to a stored long-term memory object model) are not the same thing. For instance, Sawada et al. (2011) explicitly argue that once the wireframe contour-based 3D model has been computed, it may be “wrapped” in surfaces in order that surface-based attributes (e.g., colour, texture) may be bound to shape to facilitate recognition.

Nevertheless, the current findings add constraints to such models positing edge-based reconstructions of 3D object geometry, not least because they will have to explain the current evidence that surfaces are used as image primitive for recognition performance regardless of global shape attributes, such as shape regularity. Specifically, when surface diagnosticity and shape regularity were separated in Experiment 2, surface diagnosticity determined performance: Primes containing highly diagnostic surfaces, regardless of whether they came from regular or irregular objects, led to superior recognition performance and interacted with prime type. Although it is not entirely unlikely that a regularized 3D frame model was computed for irregular objects, individual surface shape was the driver of recognition performance for irregular objects.

In summary, the current study examined the shape primitives that mediate perception and recognition of 3D objects. The results of two experiments showed an advantage for surface-based parts over closed contour fragments. This advantage interacted with surface shape diagnosticity, so that when surface shape was highly diagnostic of object identity, recognition performance was equally good regardless of whether surfaces were arranged in a volumetric or nonvolumetric configuration. The results point to the importance of surface structure in object shape representation, consistent with the hypothesis that surfaces are a powerful image primitive that can support several different kinds of tasks. This proposal is supported by recent evidence in machine vision (e.g., Lee & Park, 2002) as well as neuroimaging studies (e.g., Yamane, Carlson, Bowman, Wang, & Connor, 2008), and provides a parsimonious hypothesis for the representation of both regular and irregular objects for human object recognition.
